# Dual Catalytic Hairpin Assembly-Based Automatic Molecule Machine for Amplified Detection of Auxin Response Factor-Targeted MicroRNA-160

**DOI:** 10.3390/molecules26216432

**Published:** 2021-10-25

**Authors:** Lei Wang, Xing Dai, Yujian Feng, Qiyang Zhao, Lin Liu, Chang Xue, Langtao Xiao, Ruozhong Wang

**Affiliations:** 1 Hunan Provincial Key Laboratory of Phytohormones and Growth Development, College of Bioscience and Biotechnology, Hunan Agricultural University, Changsha 410128, China; wanglei20211015@163.com (L.W.); 18374993460@163.com (X.D.); 1326093445a@gmail.com (Y.F.); LTS14021703@gmail.com (Q.Z.); 2 Guangdong Provincial Key Laboratory of Plant Epigenetics, College of Life Sciences and Oceanography, Shenzhen University, Shenzhen 518060, China; linliu@szu.edu.cn; 3 Key Laboratory of Laboratory Medicine, Ministry of Education of China, Zhejiang Provincial Key Laboratory of Medicine Genetics, School of Laboratory Medicine and Life Sciences, Institute of Functional Nucleic Acids and Personalized Cancer Theranostics, Wenzhou Medical University, Wenzhou 325035, China

**Keywords:** miRNA160, dual catalytic hairpin assembly process, signal amplification strategy, AMM system, strand displacement reaction

## Abstract

MicroRNA160 plays a crucial role in plant development by negatively regulating the auxin response factors (ARFs). In this manuscript, we design an automatic molecule machine (AMM) based on the dual catalytic hairpin assembly (D-CHA) strategy for the signal amplification detection of miRNA160. The detection system contains four hairpin-shaped DNA probes (HP1, HP2, HP3, and HP4). For HP1, the loop is designed to be complementary to miRNA160. A fragment of DNA with the same sequences as miRNA160 is separated into two pieces that are connected at the 3′ end of HP2 and 5′ end of HP3, respectively. In the presence of the target, four HPs are successively dissolved by the first catalytic hairpin assembly (CHA1), forming a four-way DNA junction (F-DJ) that enables the rearrangement of separated DNA fragments at the end of HP2 and HP3 and serving as an integrated target analogue for initiating the second CHA reaction, generating an enhanced fluorescence signal. Assay experiments demonstrate that D-CHA has a better performance compared with traditional CHA, achieving the detection limit as low as 10 pM for miRNA160 as deduced from its corresponding DNA surrogates. Moreover, non-target miRNAs, as well as single-base mutation targets, can be detected. Overall, the D-CHA strategy provides a competitive method for plant miRNAs detection.

## 1. Introduction

MicroRNA (miRNA) is a type of non-coding single-stranded RNA widely found in eukaryotic cells, with a length of about 20–24 nucleotides [1,2]. MiRNA helps regulate various biological processes in plants, including maintenance of genome integrity, development, metabolism, alongside adaptive responses to environmental stress [3,4,5,6]. For example, miRNA160 plays an important role in the auxin (IAA) response in plants by targeting auxin response factor (ARF) genes, including ARF10, ARF16, and ARF17 genes [7,8,9]. In the auxin signal transduction pathway, miRNA160 is an essential factor for maintaining the normal development of roots, leaves, and floral organs, alongside seed germination and callus formation [10,11,12,13]. To maintain the water balance in leaves, miR160 serves as a regulator to ensure the normal development and environmental adaptation of plants [7,8]. Moreover, miR160 affects the expression of ARF17 and thus is related to tapetum development and pollen wall formation [14]. As a result, there has been enormous interest in developing biosensors for the sensitive, convenient, and cost-effective detection of miRNA160.

The detection of miRNAs remains a great challenge owing to the inherent properties, such as small size, low expression level, and high sequence homology [15,16,17,18]. In general, traditional miRNA detection methods include microarray, northern blotting, and quantitative reverse transcription PCR (RT-qRCR) [19,20,21]. Despite the advantages of microarrays, such as high-throughput and multiple miRNA detection, the demands of complicated instrumentation greatly limit the application of microarrays for the online detection of miRNA [22,23]. The time-consuming operation and limited throughout, as well as poor sensitivity, restrict the practical detection of northern blotting for trace miRNA detection [24]. Although RT-qRCR assay is accomplished with high speed and high sensitivity, the thermal-recycling controlling system and expensive instruments increase the detection cost [25,26,27]. Due to these limitations above, the development of new sensing probes is one of the current areas driving the demand for convenient, simple, sensitive, and specific detection of miRNAs.

Besides genetic molecules in living systems, DNA is now also known as a versatile tool in the fields of biochemical analysis and nanomaterials [28,29,30,31]. Due to the advantages of programmability, controllability, and diversity of base-pairing, a variety of DNA nanostructures have been assembled and found spectacular applications in designing DNA sensing probes, providing numerous opportunities for reliable detection of various target analytes, such as DNA, RNA, metal ion, and protein [32,33,34,35,36,37]. For example, Deng et al. [38] developed a rolling circle amplification (RCA)-based amplification technology for miRNA detection with high specificity. Although the method is sensitive, the synthesis and purification of the circular probe require complicated procedures. Moreover, the usage of protein enzymes inevitably increases the detection cost. To overcome those difficulties, various signal amplification strategies without protein enzymes were developed, such as hybrid chain reaction (HCR) and catalytic hairpin assembly (CHA) [39,40,41,42]. Both HCR and CHA are an enzyme-free process that provides an efficiently-amplified signal and have been often applied in the detection of miRNAs [43,44].

In this manuscript, we present an automatic molecule machine (AMM) based on the dual catalytic hairpin assembly (D-CHA) process for the signal amplification detection of ARF-targeted miRNA160. The general CHA reaction uses complementary DNA strands as DNA probes and is introduced into hairpin-shaped structures before the detection [45,46,47], providing several advantages, such as low detection cost, convenient storage, low background signal, and simple operation [48,49,50]. The CHA-based detection has been used as a satisfactory alternative amplification method for the detection of numerous biomarkers in medical diagnoses [51,52,53] and combined with different analytical techniques, such as fluorescence, electrochemistry, colourimetry, surface plasmon resonance (SPR), and electrophoresis [54,55,56,57,58]. In this work, the AMM system contains four hairpin-shaped DNA probes (HP), including HP1, HP2, HP3, and HP4. Specifically, HP1 was used as a recognition element for miRNA160. A DNA fragment with the same sequence as target miRNA160 is split into two halves and innovatively introduced at the 3′ end of HP2 and 5′ end of HP3, respectively. A pair of fluorophore (FAM) and quencher (BHQ1) molecules are modified at the ends of HP4 for signalling. After the target induces the assembly of four-way DNA junction (F-DJ) based on the first catalytic hairpin assembly (CHA), the split DNA halves are brought together and serve as an integrated target analogue to initiate the second CHA reaction. This dual-CHA-based signal amplification is termed D-CHA. The comparative experiments have demonstrated that the D-CHA strategy displays a better assay performance compared with traditional CHA, possessing several advantages, such as simple operation procedure, high sensitivity, and specificity.

## 2. Results and Discussion

### 2.1. Design of AMM Machine and Its Dual Catalytic Hairpin Assembly

**Figure 1:**
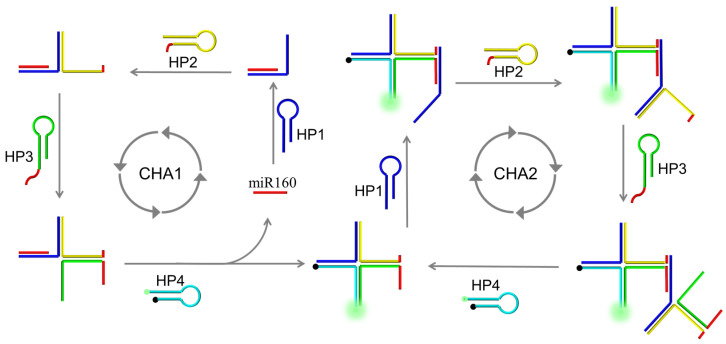
A schematic diagram of experimental principles of the AMM system for plant miRNA160 detection. The AMM system contains two catalytic hairpin assembly (CHA) processes. CHA1: In the presence of the target, HP1 is dissolved and releases the 3′ end of HP1 that hybridizes with HP2 and releases the 3′ end of HP2. Similarly, the hairpin-shaped HP3 is opened and subsequently hybridizes with HP4. The released 3′ sequence of HP4 can, in turn, bind to HP1, displacing the pre-hybridized target species and forming a four-way DNA junction (F-DJ). CHA2: Besides the released target initiating the next round of hybridization/displacement reactions, the red DNA fragments at the ends of HP2 and HP3 are brought together and serve as an integrated target analogue to initiate the second CHA reaction.

The AMM system contains four hairpin-shaped (HP) DNA probes, HP1, HP2, HP3, and HP4. The loop of HP1 is designed to be a recognition element for miRNA160. A DNA fragment with the same sequences as target miRNA160 was split into two halves and placed at the 3′ end of HP2 and 5′ end of HP3, respectively. For signalling, a pair of fluorophore (FAM) and quencher (BHQ1) molecules were modified at the ends of HP4. As shown in Figure 1, a dual catalytic hairpin assembly (D-CHA) process contains CHA1 and CHA2 for ultrasensitive detection of miRNA160. CHA1: In the presence of the target, four HPs were successively dissolved, forming a four-way DNA junction (F-DJ). Specifically, HP1 was first dissolved by the target species, releasing its 3′ end. The released 3′ sequence of HP1 is designated to bind HP2 by strand displacement, releasing the 3′ end of HP2. Similarly, HP3 and HP4 are opened, and the released 3′ sequence of HP4, in turn, hybridizes with HP1, releasing the target species and initiating the next round of hybridization/displacement reactions. CHA2: After the formation of F-DJ from CHA1, the two red halves at the ends of HP2 and HP3 are brought together and serve as a target analogue to initiate the second CHA reaction. The reaction process is the same as CHA1, but a combined DNA fragment is used as a target instead of miRNA160. The reaction continues autonomously until most DNA probes are assembled into F-DJ.

### 2.2. Feasibility of AMM for miRNA160 Detection

**Figure 2:**
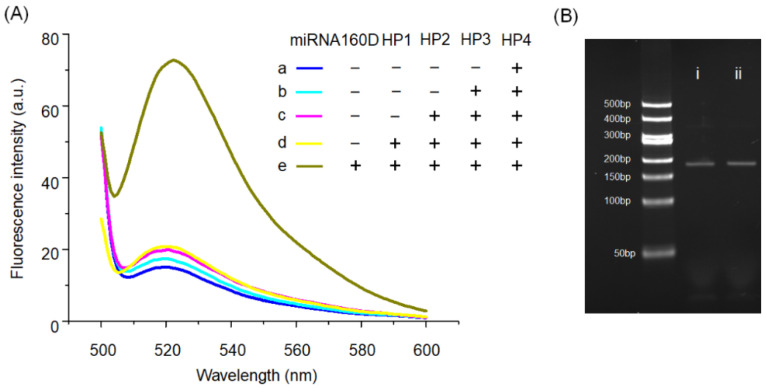
The feasibility of the AMM system for the detection of plant miRNA160D. (**A**) Fluorescence spectra of various reaction mixtures were collected to evaluate the assay ability. Sample a: HP4; Sample b: HP3 + HP4; Sample c: HP2 + HP3 + HP4; Sample d: HP1 + HP2 + HP3 + HP4; Sample e: HP1 + HP2 + HP3 + HP4 + miR160. (**B**) Comparative nPAGE analysis of reaction products of four-way DNA junction (F-DJ) from D-CHA-based system that triggered by target (i) and the reaction mixture of HP1, HP2, HP3, and HP4 after annealing (ii). The concentration of HP1, HP2, HP3, HP4, and target are 250 nM, 250 nM, 250 nM, 250 nM, and 125 nM, respectively. HP2-II and HP3-II were separately used as HP2 and HP3 in this section; the optimal process can be found in Figure S3.

Considering the intrinsic characteristics of RNA, such as susceptibility to nuclease degradation, a DNA analogue of miRNA160 (named miRNA160D) was used in this section. The feasibility of the AMM system for the miRNA160D amplification detection was validated by collecting the fluorescence spectrum. As shown in Figure 2A, the low fluorescence intensity is detected for HP4 in sample a, indicating that FAM is effectively quenched by the BHQ1 due to the hairpin-shaped structure of HP4. Subsequently, when HP3, HP2, and HP1 were successively added to HP4, no substantial increase in fluorescence intensity was detected, as shown in samples b, c, and d, and demonstrated that the four hairpin-shaped DNA probes could stably coexist in the absence of target miRNA. In contrast, the high fluorescence intensity can be detected once the target is added to sample e, revealing that AMM can provide an intense signal response. The data in Figure 2B shows that the F-DJ prepared from the D-CHA-based system triggered by target (i) and the reaction mixture (ii) of HP1, HP2, HP3, and HP4 after annealing have identical gel mobility. Overall, the low background and the high signal response of the AMM system demonstrate that it is suitable for the target detection with a low expression, such as miRNAs.

### 2.3. D-CHA-Based Signal Amplification

For improving the high sensitivity of the sensing system, the dual catalytic hairpin assembly (D-CHA) strategy was proposed. After the first CHA reaction, F-DJ is formed and brings two halves of the target analogue at the ends of the HP2 and HP3 together, initiating the second CHA reaction and providing additional stimuli that contribute to ultrasensitive miRNA160D detection.

**Figure 3:**
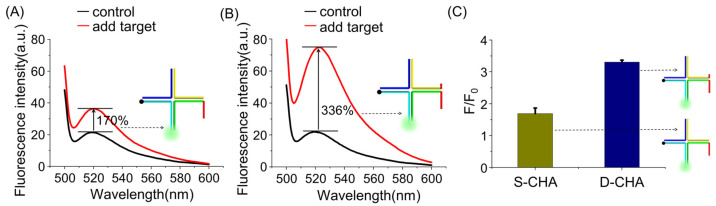
The assay performance of S-CHA-(**A**) and D-CHA-(**B**) based system. The red line and black line denote the fluorescence spectra of the system in the presence and absence of the target, respectively. (**C**) Comparative evaluation of the signal-to-noise ratio (F/F0) of the two systems for miRNA160D detection. F0 and F represent the signal in the absence and presence of target miR160, respectively. The concentration of HP1, HP2, HP3, HP4, and target are 250 nM, 250 nM, 250 nM, 250 nM, and 125 nM, respectively. The error bars represent means ± SD (*n* = 3).

To provide convincing evidence for the D-CHA amplification effect, the closely-related comparative experiments were performed. A CHA-based system with the same component was used as control, but HP2-VI without the extended end was used instead of HP2. After the first CHA reaction, the four-way DNA junction (F-DJ) is formed, and the second CHA cannot be triggered owing to the lack of a combined target analogue. The corresponding assay system is called a single CHA-based one (S-CHA). As shown in Figure 3, only a 170% fluorescence increase is detected for S-CHA in the presence of target miRNA, while the fluorescence intensity of D-CHA increases by 336% upon target stimulation, demonstrating that the combined target analogue efficiently contributes to the signal amplification. Moreover, the fluorescence increase of the D-CHA-based system is much higher than S-CHA, indicating that the design of D-CHA does promote the autocatalytic reaction acceleration (See Supplementary Materials Figure S1).

### 2.4. Assay Performance of the AMM System for miRNA160D Detection

To make AMM operate with high efficiency in the presence of miRNA160D, the reaction time and probe sequences were first optimized. To investigate the optimal reaction time, the fluorescence intensity of AMM in the presence and absence of miRNA160D was recorded after incubating for different time periods ranging from 0.5 to 10 h. As exhibited in Figure S2, the signal-to-noise ratio (F/F0) gradually increases with the reaction time progresses until reaching the plateau. It can be found that the optimal time is 8 h. The sequences of two halves of the target analogue at the ends of HP2 and HP3 are crucial for the D-CHA reaction. Thus, the dependence of the fluorescence signal on the sequences of HP2 and HP3 was studied by using different HP2 and HP3 for miRNA160D detection. As shown in Figure S3, HP2-II and HP3-II offered the highest signal-to-noise ratio (F/F0) and was thus used in the subsequent experiments.

**Figure 4:**
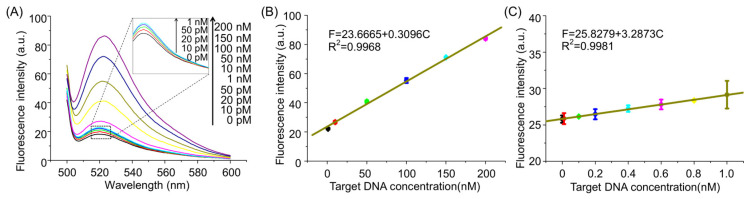
Quantitative detection of plant miRNA160D by AMM. (**A**) Fluorescence spectra of AMM upon the different concentrations of target DNA: 0, 0.01 nM, 0.02 nM, 0.05 nM, 1 nM, 10 nM, 30 nM, 50 nM, 80 nM, 100 nM, 150 nM, and 200 nM. Inset: Fluorescence spectra at low concentrations of miRNA160D. The calibration curves in the high concentration range (**B**) and low concentration range (**C**). The concentration of HP1, HP2, HP3, and HP4 are 250 nM, 250 nM, 250 nM, and 250 nM, respectively. The error bars represent means ± SD (*n* = 3).

Subsequently, the assay performance of the AMM system was evaluated by probing a series of different concentrations of miRNA160D under the optimized experimental conditions. As shown in Figure 4A, the signal intensity at 520 nm gradually increases with the increase in target concentration, demonstrating that the quantitative detection of miRNA160D can be achieved. The fluorescence spectra of AMM in the presence of a low concentration of target miRNA can be found in the inset. If the target concentration is capable of generating a detectable signal higher than the background, it is defined as the limit of detection (LOD), the LOD as low as 10 pM is achieved for miRNA160D detection. Figure 4B,C plots the fluorescence intensity against the target concentration in the high concentration range and low concentration range, where F and C represent the peak fluorescence intensity and the miRNA160D concentration, respectively. Meanwhile, a detectable signal can be obtained in the presence of a synthetic RNA target (Figure S4) or extracted RNA from peach (Figure S5), indicating the feasibility of the method for RNA detection.

### 2.5. Detection Specificity

**Figure 5:**
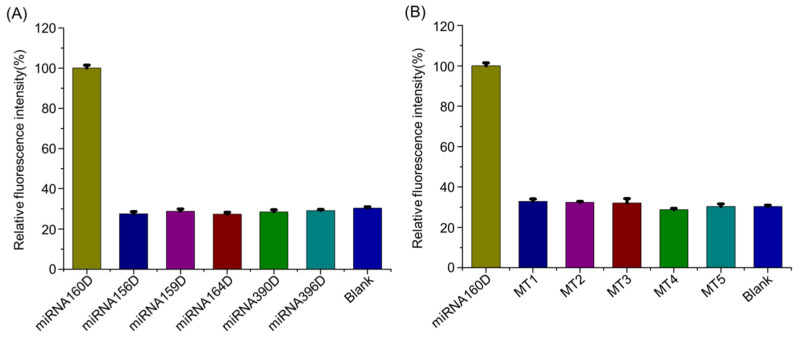
The detection specificity of AMM towards miR160. The fluorescence intensity was recorded upon non-targets (**A**) and mutant targets with one-, two-, three-, four-, and five-base mismatched targets (abbreviated as MT1, MT2, MT3, MT4, and MT5, respectively) (**B**). The concentration of HP1, HP2, HP3, HP4, and target are 250 nM, 250 nM, 250 nM, 250 nM, and 125 nM, respectively. The error bars represent means ± SD (*n* = 3).

The selectivity is another essential parameter for evaluating the assay performance of DNA probes. To test the selectivity of the AMM machine, the DNA surrogates of common plant miRNAs (miRNA156D, miRNA159D, miRNA164D, miRNA390D, miRNA396D) were detected against miRNA160D under identical conditions. As exhibited in Figure 5A, if defining the signal intensity induced by miRNA160D is 100%, the relative fluorescence signal of non-target miRNAs is not more than 30%, demonstrating a high detection specificity of AMM towards target miRNA. Moreover, five mutant targets were retrieved from the miRBase database (http://www.mirbase.org/) (accessed on 10 September 2021), including one-, two-, three-, four-, and five-base mismatched target (MT1, MT2, MT3, MT4, and MT5), were detected. As depicted in Figure 5B, no substantial signal increase is observed upon mutant targets compared with the background. The feasibility of AMM for discriminating the mutant miRNA from miRNA160D has also been demonstrated via a blind test (Table S2). Namely, AMM is able to discriminate the single-base-mismatched targets, indicating a high capability to distinguish mismatched targets. Besides, compared with previous CHA-based probes, AMM displays comprehensive advantages (Table S3).

## 3. Conclusions

In summary, a dual CHA amplification is proposed on the basis of the extension of hairpin probe ends by adding the same sequences as the target, leading to the formation of an automatic molecule machine (AMM) system for the sensitive detection of ARF-targeted miRNA160. In the presence of the target, four HPs are successively dissolved during the first CHA, generating a four-way DNA junction (F-DJ) and releasing the pre-hybridized target species. The next round of reactions is initiated by the released target; F-DJ has a combined target analogue composed of two halves that are respectively designed at the end of HP2 and HP3, enabling another CHA reaction. Thus, the amplified fluorescence signal can be achieved compared with traditional CHA. The target can be detected down to 10 pM. Moreover, the non-target miRNA, as well as single-base mutation targets, can be distinguished, indicating a high specificity. Overall, the AMM system comes with simple operation steps, high sensitivity, and specificity, providing a competitive method for detecting plant miRNA.

## 4. Materials and Methods

### 4.1. Materials

DNA sequences used in this experiment were synthesized and purified by Shanghai Shengong Biological Engineering Co., Ltd. (Shanghai, China). All DNA sequences are listed in Table S1. HP4 modified with FAM and BHQ1 was purified by high-performance liquid chromatography (HPLC), while other label-free sequences were polyacrylamide gel electrophoresis (PAGE)-purified. The DNA sequences were dissolved in ultrapure water to a concentration of 10 µM after centrifugation at 8000 rpm for 2 min, and the resulting solutions were kept at 4 °C before use. Other reagents were analytical grade and purchased from Sinopharm Chemical Reagent Co. (Shanghai, China).

### 4.2. Preparation of AMM System

Four DNA probes (HP1, HP2, HP3, and HP4) were separately annealed to form a hairpin-shaped structure by following steps: 1 μL of 10 μM DNA probe was added into 9 μL of TAE buffer (2 mM EDTA, 12.5 mM MgCl_2_, 40 mM Tris, pH = 8.0) and heated at 90 °C for 5 min. The resulting solution was gradually cooled to room temperature. Subsequently, AMM (40 μL) was prepared by mixing the four solutions together.

### 4.3. RNA Extracted from Peach

The mortar and pestle were first cooled sufficiently by liquid nitrogen. The peach kernel sample was added and ground to powder. After the liquid nitrogen was completely volatilized, 1 mL of Trizol was added and reacted for 10 min (the sample volume should not exceed 10% of Trizol). Chloroform (0.2 mL) was added and reacted for another 10 min. Afterwards, the supernatant was collected by centrifuging (15,800 rpm) at 4 °C for 10 min. Isopropanol (0.5 mL) was added and reacted at −20 °C for at least 15 min, and then the RNA precipitation was collected by centrifuging (15,800 rpm) at 4 °C for 10 min. The RNA was washed by cooled ethyl alcohol (1 mL, 75%), then resuspended in diethyl oxydiformate (DEPC) treated water.

### 4.4. Fluorescence Measurement

A 0.5-μL aliquot of the target at a given concentration of H_2_O was added to the AMM system prepared above, followed by incubating at 25 °C for 8 h. After diluting with TAE buffer to the final volume of 200 μL, the fluorescence spectrum of the resulting solutions was collected between 500 to 600 nm on a Hitachi F-7000 (Hitachi Ltd., Tokyo, Japan). The fluorescence peak at λem = 520 nm was used to evaluate the fluorescence intensity.

## Data Availability

Data regarding this article will be provided upon request.

## Supplementary Materials

The following are available online, Figure S1: The real-time monitoring of fluorescence change; Figure S2: Optimization of reaction time; Figure S3: The dependence of the fluorescence signal on the sequence of HP2 and HP3; Figure S4: Fluorescence spectra of AMM upon the different concentrations of target RNA; Figure S5: The detection of miRNA160 that was extracted from the peach; Table S1: Oligonucleotide sequences used in this work; Table S2: Blind test results of miRNA160D; Table S3: Comparison in assay ability and advantages [59,60,61,62,63].

## Sample Availability

Samples of the compounds are available from the authors.
