# Supplementary material for: Dual Catalytic Hairpin Assembly-Based Automatic Molecule Machine for Amplified Detection of Auxin Response Factor-Targeted MicroRNA-160
Source: Molecules. 2021 Oct 25;26(21):6432. doi: 10.3390/molecules26216432 (PMC8588017; doi:10.3390/molecules26216432)
Supplement: Supplementary file 1 [file molecules-26-06432-s001.zip › molecules-1364904-supplementary.pdf]

Supplementary Materials

Dual Catalytic Hairpin Assembly-based Automatic Molecule Machine for Amplified Detection of Auxin Response Factor-targeted MicroRNA-160

Lei Wang[1], Xing Dai[1], YuJian Feng[1], Qiyang Zhao[1], Lin Liu[2], Chang Xue,[3,] * Langtao Xiao[1,]*, Ruozhong Wang[1,]*

[1] Hunan Provincial Key Laboratory of Phytohormones and Growth Development, College of Bioscience and Biotechnology, Hunan Agricultural University, Changsha 410128, China.

[2] Guangdong Provincial Key Laboratory of Plant Epigenetics, College of Life Sciences and Oceanography, Shenzhen University, Shenzhen 518060, China.

[3] Key Laboratory of Laboratory Medicine, Ministry of Education of China, Zhejiang Provincial Key Laboratory of Medicine Genetics, School of Laboratory Medicine and Life Sciences, Institute of Functional Nucleic Acids and Personalized Cancer Theranostics, Wenzhou Medical University, Wenzhou 325035, China.

* Corresponding Author: wangruozhong@hunau.edu.cn, changxue@wmu.edu.cn, ltxiao@hunau.edu.cn

Table S1. Oligonucleotide sequences used in this work. [a]

Function and name of probes		Sequence(5'-3')
Hairpin-shaped DNA probes (HP)	HP1	TGGCATACAGGGAGCCAGGCACCGATGCCACGGTGCCTGGCTCCCTGT
	HP2-I	ACAGGGAGCCAGGCACCGTGGCATCCCTGTATGCCACGGTGCCTGGCTTGCCA
	HP2-II	ACAGGGAGCCAGGCACCGTGGCATCCCTGTATGCCACGGTGCCTGGCTATGCCA
	HP2-III	ACAGGGAGCCAGGCACCGTGGCATCCCTGTATGCCACGGTGCCTGGCTTATGCCA
	HP2-IV	ACAGGGAGCCAGGCACCGTGGCATCCCTGTATGCCACGGTGCCTGGCTCTGTATGCCA
	HP2-V	ACAGGGAGCCAGGCACCGTGGCATCCCTGTATGCCACGGTGCCTGGCTCCTGTATGCCA
	HP2-VI	ACAGGGAGCCAGGCACCGTGGCATCCCTGTATGCCACGGTGCCTGGCT
	HP3-I	TGCCTGGCTCCCTGTAAGCCAGGCACCGTGGCATACAGGGCTGGCTCCCTGTATGCCACGGTGC
	HP3-II	TGCCTGGCTCCCTGTAGCCAGGCACCGTGGCATACAGGGCTGGCTCCCTGTATGCCACGGTGC
	HP3-III	TGCCTGGCTCCCTGAGCCAGGCACCGTGGCATACAGGGCTGGCTCCCTGTATGCCACGGTGC
	HP3-IV	TGCCTGGCTCCAGCCAGGCACCGTGGCATACAGGGCTGGCTCCCTGTATGCCACGGTGC
	HP3-V	TGCCTGGCTCAGCCAGGCACCGTGGCATACAGGGCTGGCTCCCTGTATGCCACGGTGC
	HP4	FAM-GCACCGTGGCATACAGGGAGCCAGCGGTGCCTGGCTCCCTGTATGCCA-BHQ1
Target or non-target species	miR160D	TGCCTGGCTCCCTGTATGCCA
	miR160	UGCCUGGCUCCCUGUAUGCCA
	MT1	TGCCTGGCTCCCTGAATGCCA
	MT2	TGCTCGGCTCCCTGTATGCCA
	MT3	TGCCTGGCTCCCTGGATGCCA
	MT4	CGCCTGGCTCCTTGTATGCCA
	MT5	CGCCTGGCTCCCTGCATGCCG
	miR156D	TGACAGAAGAGAGTGAGCAC
	miR159D	GAGCTCCCTTCCTCCAAAACG
	miR164D	TGGAGAAGCAGGGCACGTGCA
	miR390D	AAGCTCAGGAGGGATAGCGCC
	miR396D	TTCCACAGCTTTCTTGAACTT

[a]The boxed fragments in each DNA probes represent the complementary sequences capable of forming a hairpin structure via intramolecularly hybridization. The bases in red in target and non-target species indicate the point mutations.

Table S2. Blind test results of miRNA160D.

Samples	RFI（%）	RSD[I]（n=3）[II]（%）	Output	Marked
WT1	33.1	3.6	Non-target	MT4
WT2	33.2	6.1	Non-target	MT5
WT3	100	1.0	miRNA160	miRNA160
WT4	34.9	2.1	Non-target	MT3
WT5	35.6	4.2	Non-target	MT2
WT6	39.8	2.0	Non-target	MT1

[I]RSD represents relative standard deviation

[II]Samples were assayed in triplicate

Table S3. Comparison in assay ability and advantages between D-CHA strategy and previous CHA-based probes.[a]

Material	Target	LOD(pM)	Enzyme-free process	Ref.
MnO_2 nanosheets + DNA	miRNA	330	yes	(1)
AuNPs + DNA	nucleic acid	25	yes	(2)
DNA	miRNA	58.1	yes	(3)
DNA	miRNA	9.2×10^{-3}	no	(4)
Biotin + DNA	protein	6.5	no	(5)
DNA	miRNA	10	yes	This work

[a]Comparative discussion:

In Ref. 1 to 3, these three CHA-based probes were developed for detection of nucleic acid target including miRNAs. Clearly, the assay ability that based on traditional CHA needs to be improved, such as the limit of detection (LOD). Moreover, in Ref. 1 and 2, the preparation of probes are complicated and time-consuming.

In Ref. 4 and 5, satisfactory LOD was obtained by enzyme-aided recycling amplification, however, these methods are hampered by the susceptibility of enzyme in the complex intracellular environment and also increase the assay cost.

In conclusion, D-CHA strategy provides a high sensitive and enzyme-free detection, displaying comprehensive advantages.

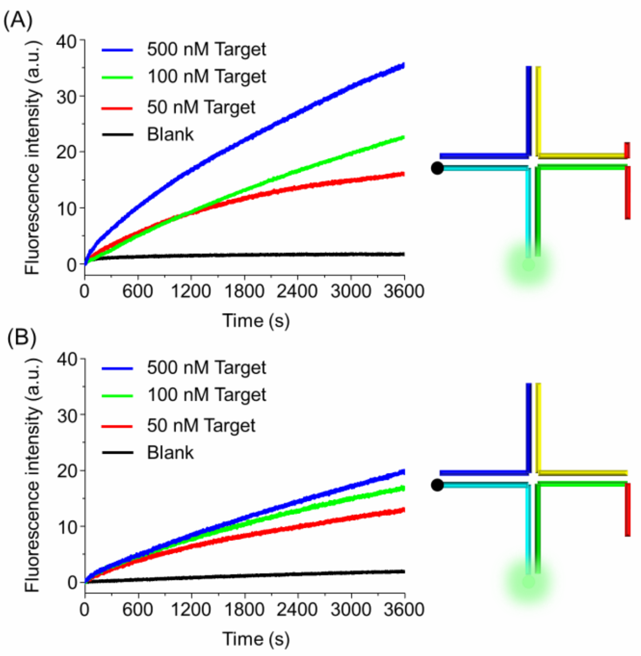

Figure S1. The real-time monitoring of fluorescence change of D-CHA-(A) and S-CHA- (B) based system upon addition of target molecule. The concentration of HP1, HP2, HP3, HP4, and target are 250 nM, 250 nM, 250 nM, 250 nM, and 125 nM, respectively. The error bars represent means ± SD (n = 3).

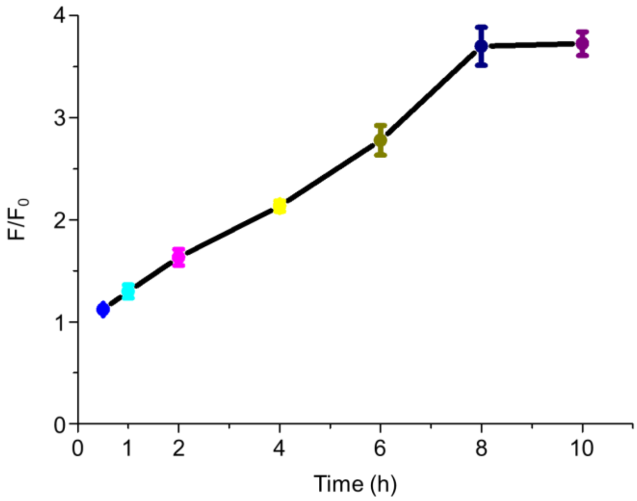

Figure S2. Optimization of reaction time. The signal-to-noise ratio (F/F_0) of AMM for miRNA160 detection after incubating for different time periods ranging from 0.5 to 10 h. F is the fluorescence intensity of AMM system in the presence of target, while F_0 represents the signal in the absence of target miR160. The concentration of HP1, HP2, HP3, HP4, and target are 250 nM, 250 nM, 250 nM, 250 nM, and 125 nM, respectively. The error bars represent means ± SD (n = 3).

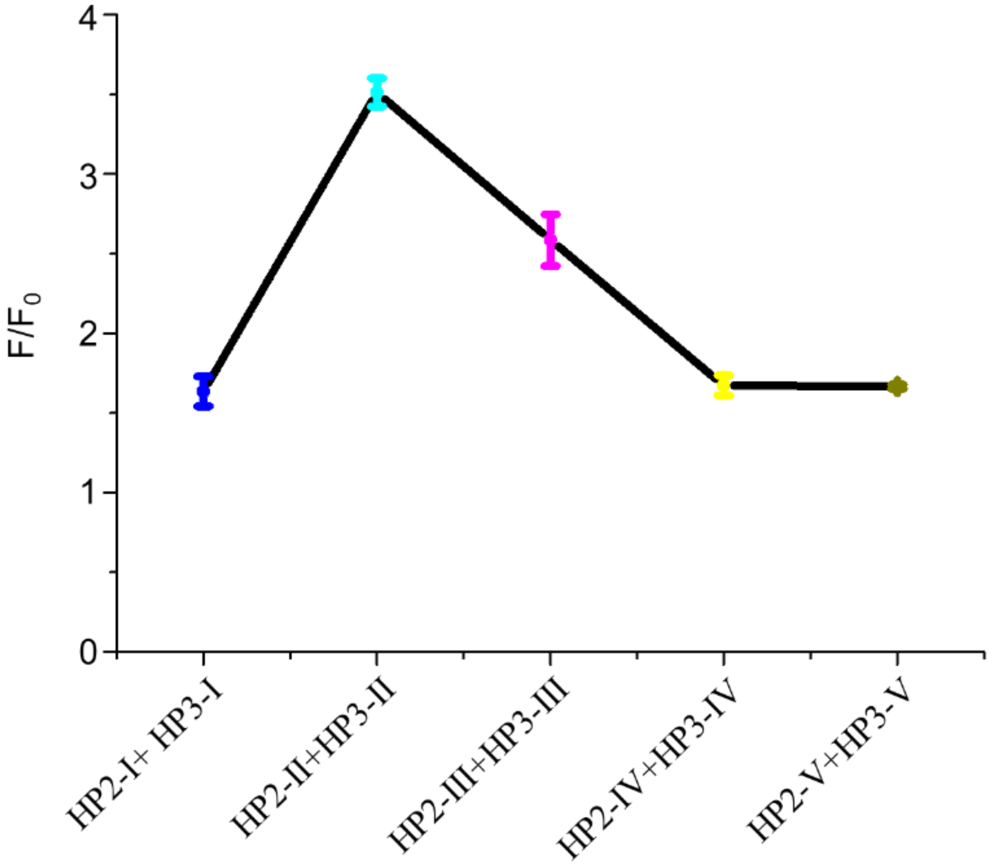

Figure S3. The dependence of fluorescence signal on the sequence of HP2 and HP3. The signal-to-noise ratio (F/F_0) of AMM for miRNA160 detection by using different combination of HP2 and HP3. F is the fluorescence intensity of AMM system in the presence of target, while F_0 represent the signal in the absence of target miR160. The concentration of HP1, HP2, HP3, HP4, and target are 250 nM, 250 nM, 250 nM, 250 nM, and 125 nM, respectively. The error bars represent means ± SD (n = 3).

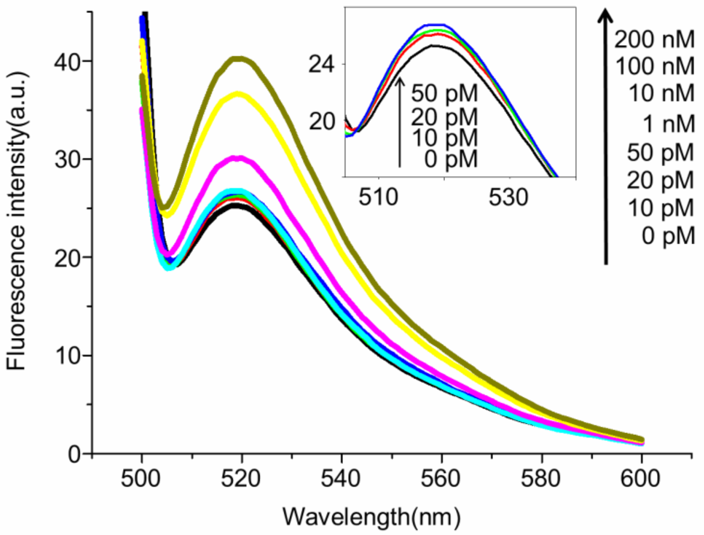

Figure S4. Fluorescence spectra of AMM upon the different concentrations of target RNA: 0, 0.01 nM, 0.02 nM, 0.05 nM, 1 nM, 10 nM, 100 nM, and 200 nM. Inset: Fluorescence spectra at low concentration of miRNA160.

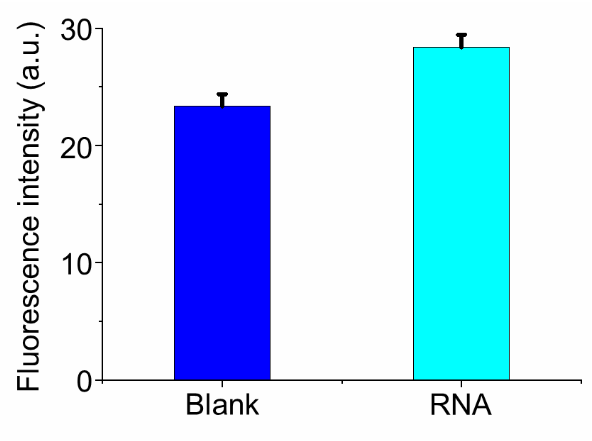

Figure S5. The detection of miRNA160 that extracted from peach. The error bars represent means ± SD (n = 3).
